# Menopause and the influence of culture: another gap for Indigenous Australian women?

**DOI:** 10.1186/1472-6874-12-43

**Published:** 2012-12-12

**Authors:** Emma K Jones, Janelle R Jurgenson, Judith M Katzenellenbogen, Sandra C Thompson

**Affiliations:** 1 Faculty of Medicine and Dentistry, The University of Western Australia, Stirling Highway, Perth Western, Australia; 2 Combined Universities Centre for Rural Health, University of Western Australia, Geraldton, Western Australia

**Keywords:** Menopause, Indigenous, Aboriginal Attitudes, Perceptions, Experiences, Culture

## Abstract

**Background:**

There is great variation in experience of menopause in women around the world. The purpose of this study was to review current understanding of Australian Aboriginal and Torres Strait Islander (Indigenous) women’s experiences of menopause. The literature pertaining to the perception, significance and experience of menopause from a number of cultural groups around the world has been included to provide context for why Indigenous women’s experience might be important for their health and differ from that reported in other studies of Australian women and menopause.

**Methods:**

A search of databases including Ovid Medline, Pubmed, Web of Science, AUSThealth, AMED, EMBASE, Global Health and PsychINFO was undertaken from January 2011 to April 2011 using the search terms menopause, Indigenous, Aboriginal, attitudes, and perceptions and repeated in September 2012.

**Results:**

Considerable research shows significant variation across cultures in the menopausal experience. Biological, psychological, social and cultural factors are associated with either positive or negative attitudes, perceptions or experiences of menopause in various cultures. Comparative international literature shows that neither biological nor social factors alone are sufficient to explain the variation in experiences of the menopausal transition. However, a strong influence of culture on the menopause experience can be found. The variation in women’s experience of menopause indicates that different cultural groups of women may have different understandings and needs during the menopausal transition. While considerable literature exists for Australian women as a whole, there has been little investigation of Australian Indigenous women, with only two research studies related to Indigenous women’s experiences of menopause identified.

**Conclusions:**

Differences in biocultural experience of menopause around the world suggest the importance of biocultural research. For the Indigenous women of Australia, the relative contribution of culture, social disadvantage and poor general health compared with non-Indigenous women to the experience of menopause is unknown. As such, further research and understanding of the experience of Indigenous women around Australia is needed. This information could assist individuals, families, cultural groups and healthcare providers to enhance management and support for Indigenous Australian women.

## Background

All women that live long enough will experience a biological decline in ovarian function leading to reproductive senescence marking the end of their fertility and capacity for reproduction. However, the actual experience of menopause for women is individual and like health is influenced by a multitude of biological, cultural, socioeconomic and lifestyle factors [1-9]. Offering the best advice and care possible for women during this time, requires a good understanding of the factors that play a role in influencing the menopausal experience. This review of current literature examines some of the evidence in various cultural groups around the world and the influence of culture on the menopausal experience before focusing on current literature published on Australian Aboriginal and Torres Strait Islander (hereafter Indigenous) women’s experience of menopause.

The menopausal transition is defined biologically as *beginning with variations in menstrual cycle length and a monotropic rise in FSH, and ending with the final menstrual period, confirmed only when followed by 12 months of amenorrhoea*.[10] Menopause usually occurs between 45 and 54 years of age with an average onset of 51 years [11]. A number of negative health outcomes occur more frequently after menopause, including osteoporosis [12,13], loss of protective effect against cardiovascular disease [13], and a 60% increase in risk of metabolic syndrome [14].

Prior to menopause, women have been found to have a lower risk of cardiovascular disease than men, a finding attributed as due to the protective effects of estrogen [15]. However, research on cardiovascular disease in Indigenous and non-Indigenous people in Western Australia found that Indigenous women did not appear to experience the premenopausal protective effect from cardiovascular disease evident in non-Indigenous women [16]. Examination of current literature exposed numerous articles detailing the different menopausal experience of women in countries such as Japan, North America and Europe [4,7-9,17-30] but highlighted a major gap within the literature on the experience in Australian Indigenous women. Hence documented findings of the menopausal experiences in different cultures around the world and corresponding influential factors can be used to hypothesise and extrapolate the menopausal experience in Australian Indigenous Women.

### Aboriginal and Torres Strait Islander People of Australia

An overview of the general health status of Aboriginal and Torres Strait Islanders of Australia (collectively the Indigenous population of Australia) is important in understanding what factors may impact menopausal experience. Indigenous Australians make up 2.5% of the Australian population [31], but their burden of disease is two and half times greater than the non-Indigenous population [31]. Prevalence of health risk factors among this population is also greater with twice the rate of smoking [31], higher levels of stressful life events [31], poorer nutrition [32], and lower physical activity levels [32]. Furthermore, Indigenous women are 1.5 times more likely to be overweight or obese when compared to non-Indigenous Australians [31]. The fertility rate of Indigenous women in 2006 was 2.1 children per woman, compared to 1.8 for the wider Australian population [31], Indigenous women’s age of first child is five years younger than that of non-Indigenous Australians at 25 years old [31]. The most common contraceptive practice use in Indigenous women are the use of condoms, followed closely by the contraceptive pill [31]. However, rates of both forms of contraception become much lower the more remote the location. In these remote areas the use of contraceptive injections and implants are the preferred choice for contraception [31].

Indigenous women also have lower educational attainments when compared to non-Indigenous; 20% of Indigenous females have a post-school qualification compared to 37% for non-Indigenous women [32]. As a likely consequence of the lower education attainment, unemployment in Indigenous Australians is three times the rate of non-Indigenous Australians [31].

### Menopause

A longitudinal study put the average age of menopause in Australian women as 52.9 years [33], with the onset ranging anywhere between 45–64 years of age [33]. A higher median age for menopause has been noted as occurring in societies that have higher standards of living [34].

Symptoms of menopause can be categorised into three broad groups recognised around the world: somatic, psychological and urogenital [35] (Table 1). The only symptoms that can be directly associated with a decrease in oestrogen levels are vasomotor effects (hot flushes/flashes and night sweats), vaginal dryness, and insomnia, with the latter via the indirect effects of hot flushes and night sweats interrupting sleep [36].

**Table 1:** Categories of symptoms and prevalence in Australia

Groups of Menopause symptoms * *symptoms directly related to decline in estrogen levels*		**Prevalence in Australia Menopausal women (variable)**
**Somatic**	*Hot flushes	45-80%^37^
	*Night Sweats	38-82%^37^
	Heart discomfort	30% (Palpitations)^11^
	Sleeping problems - *insomnia	-
	Muscle and joint problems	-
**Psychological**	Depressive Mood	-
	Irritability	-
	Anxiety	-
	Physical and Mental exhaustion	-
**Urogenital**	Sexual problems	-
	Bladder problems	-
	*Vaginal dryness	45% ^11^
		Total Urogenital = 60%^11^

Given the great variation in the experience of menopause, increasing knowledge and awareness of which cultural groups are more likely to experience different symptoms and perceptions would assist these women and their health care providers with management and support of menopause when and if symptoms are experienced. Furthermore, by addressing the menopause experience in minority groups experiencing a health disparity such as Australian Indigenous women, we are in a better position to both guide future research and help improve their quality of life.

## Methods

**Figure 1:**
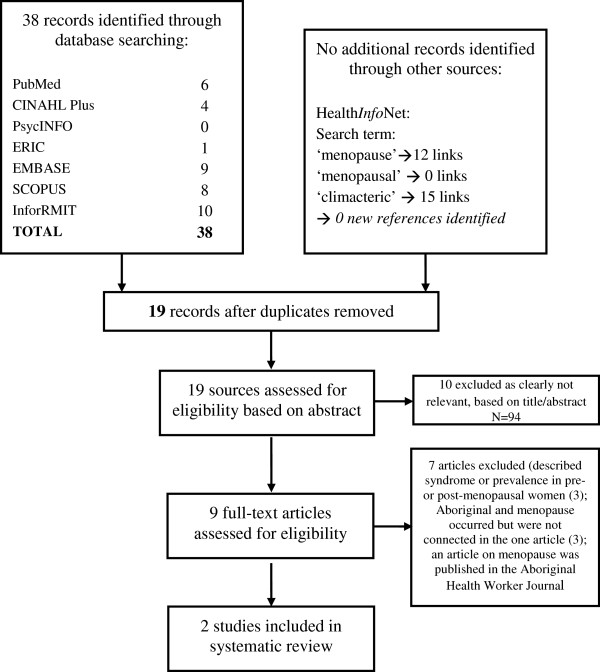
Flow of search strategy for Indigenous Australian women’s experience of menopause as per PRISMA guidelines.

The following databases were searched for relevant literature: AUSThealth, AMED (Allied and Complementary Medicine 1985 – January 2011), EMBASE Classic and EMBASE (1947 – 2001 week 2), Global Health (1910 – December 2010), Ovid Medline (1948 – January week 1 2011), PsychINFO (1806 – January week 2 2011), Pubmed (1966 – Jan 2011), Google Scholar, and the Australian Indigenous Health InfoNet. A cited reference search using Web of Science was also completed. The search was initially undertaken from January 2011 to April 2011 and repeated in September 2012 using the terms shown in Table 2. Using terms such as end of monthlies; change of life; climacteric; cessation of menstruation; The Change; midlife change; life transition; and rite of passage did not increase the number of relevant articles identified. Articles were excluded if they were not in English, or the focus of the article was not on attitudes or perceptions of menopause or primarily about an Indigenous populations’ experience of menopause. Only a limited number of articles concerning the symptoms of menopause were included, those that related to Indigenous populations or the Australian population (Figure 1). While there were studies that compared the general Australian women’s experience of menopause to other non-Western cultures, only two pieces of literature, one an unpublished thesis, were directly related to the Australian Indigenous women’s experience of menopause were identified [37].

**Table 2:** Systematic search strategy for Aboriginal and Torres Strait Islander women and menopause

**Database**	**Search Terms**	**Number of Articles Retrieved**
**PUBMED**	(Oceanic Ancestry Group [mh] OR indigenous OR aborigin* OR torres strait islander OR torres strait islanders)	6
	AND	
	(australia OR australia*)	
	AND	
	(menopause OR menopausal OR premenopause OR premenopausal OR perimenopause OR perimenopausal OR postmenopaus* OR postmenopaus* OR climacteric or climacteri* OR “change of life” or “hot flashes”)	
**CINAHL PLUS**	(MH "Climacteric+") OR (MH "Menopause+") OR (MH "Perimenopausal Symptoms+") OR (MH "Perimenopause") OR (MH "Hot Flashes") OR (MH "Postmenopausal Disorders") OR (MH "Postmenopause") OR (MH "Premenopause") OR (MH "Menopause, Premature") OR menopaus* OR premenopaus* OR postmenopaus* OR climacteri* OR “change of life” OR “hot flashes”	4
	AND	
	(australia OR Australia*)	
	AND	
	(MH “Indigenous peoples+” OR MH “Indigenous health+” OR indigenous OR aborigin* OR "torres strait islander" OR "torres strait islanders")	
**PsycINFO 1806 to August Week 4 2012**	(exp Menopause/ or menopaus$ or premenopaus$ or perimenopaus$ or postmenopaus$ or climacteri$ or change of life or hot flashes)	0
	AND	
	australia and (exp indigenous populations/ or aborigin$ or indigenous or torres strait islander$)	
**ERIC**	(menopaus$ or premenopaus$ or perimenopaus$ or postmenopaus$ or climacteric or change of life or hot flashes)	1
	AND	
	australia and (exp indigenous populations/ or aborigin$ or indigenous or torres strait islander$)	
**SCOPUS**	TITLE-ABS-KEY(menopaus* OR premenopaus* OR perimenopaus* OR postmenopaus* OR climacteri* OR “change of life” OR “hot flashes” )	8
	AND	
	TITLE-ABS-KEY(australi*)	
	AND	
	TITLE-ABS-KEY(indigenous OR aborigin* OR "torres strait islander" OR "torres strait islanders")	
**EMBASE Classic + EMBASE 1947 to 2012 September 05**	(exp early menopause/ OR exp menopause/ OR exp menopause related disorder/ OR exp "menopause and climacterium"/ OR menopaus$ OR premenopaus$ OR perimenopaus$ OR postmenopaus$ ORr climacteric$ OR change of life OR hot flashes )	9
	AND	
	(exp australia/ or australia$)	
	AND	
	(exp indigenous people/ OR exp aborigine/ OR aborigin$ OR indigenous OR torres strait islander$))	
**INFORMIT (‘Multiple databases’ search)**	(menopaus* OR premenopaus* OR perimenopaus* OR postmenopaus* OR climacteri* OR “change of life” OR “hot flashes” )	10
	AND	
	(australia OR australian)	
	AND	
	(indigenous OR aborigin* OR "torres strait islander" OR "torres strait islanders")	

*The terms 'premenopausal' and 'postmenopausal' were included to maximise 'sensitivity', since few relevant articles were retrieved in preliminary searches*.

## Results

### Factors that influence menopausal experience

There are a range of factors which have been identified from the literature search contributing to the variation in the menopausal experience. These include biological and reproductive factors, psychological, cultural and social factors.

#### Biological and reproductive factors

Women with a higher body mass index often have an increased incidence of vasomotor symptom reporting [1,4,38], although this has not been found in all studies [33]. In contrast, women who have high levels of physical activity have lower incidence [5]. Lifestyle choices such as smoking and alcohol consumption have also been linked with higher rates of hot flushes [1]. In addition, smoking reduces the age of onset of menopause by approximately three years [36] as well as increasing the incidence of symptom reporting [8,28,39]. Reproductive factors also appear to influence both the onset of menopause and the incidence of menopausal symptoms. Those consuming a diet containing high levels of phytoestrogens, common in Asian countries, report reduced incidence of hot flushes [39].Women who are nulliparous or have low parity, have spent limited or no time breastfeeding and had low use of oral contraceptives, have reported an earlier onset of menopause [4]. In comparison women of high parity, long duration of breastfeeding and long duration of oral contraceptive use have a lower incidence of menopausal symptom reporting [8].

#### Psychological factors

Individual attitudes towards menopause have a significant role in the overall experience of menopause, acting largely as self-fulfilling prophecies. Women who have negative attitudes towards menopause and/or ageing are more likely to report a greater number and frequency of menopausal symptoms [3,40-42].

Attitudes are most commonly influenced by female relatives and friends, particularly mothers [43]. However, attitudes towards menopause are not inflexible, but appear to be influenced by the stage of the menopause transition. For example postmenopausal women tend to have a more positive attitude towards menopause than premenopausal women [2,7,44]. The most negative attitudes towards menopause come from women who have undergone surgical menopause [42].

Many women have neutral perceptions of menopause or perceive it as a positive experience as it offers relief from the worries of menstruation, freedom from the necessity of contraception, and increased sexual freedom[2,20,43]. Women who place a high priority on fertility are inclined to have more negative attitudes towards menopause [39] and also women who reach menopause before achieving the number of children they desire [36]. This can occur in cultures that value women in terms of her ability to procreate [23]. In comparison, Western societies which do not necessarily place a high importance on procreation often place a high value on youthfulness. Consequently, in these societies the menopause signals age progression and loss of youth combined with loss of sexual attractiveness and can lead to negative attitudes towards the transition [36,38,45,46].

#### Social factors

The social context in which a woman lives is important to her understanding of the menopausal transition. When looking at different countries, variations in symptom reporting can be attributed to language differences, culturally shaped expectations about menopause, culturally influenced gender roles and socioeconomic status [39].

Language used to communicate a subject can demonstrate how a society perceives a topic. Menopause in the Western world it is a topic that is largely medicalised with much of the language being dominated by negative imagery such as “reproductive failure or ovarian failure” [2]. This implies that menopause is a disease state that must be treated rather than a natural biological transition. [6,36,43,45-47] In the Arab world, the word corresponding to the menopausal and midlife period means ‘desperate age’ [7], implying a pessimistic outlook towards menopause. Native American Indian women do not have a is single word for menopause[20] and the Japanese have no equivalent word for the English phrase ‘hot flush’ [8], which could indicate the relative unimportance of the symptoms or subject to daily discourse.

How a culture views menstruation has important implications for the significance of its cessation. In some cultures menstruating women are seen as impure, thus their cessation can correspond to an increased status within the community [43]. In Islamic and most African societies postmenopausal women no longer have to observe strict gender roles [45]. The women of these societies appear to have lower reporting of symptoms, possibly due to the positive role changes associated with the menopausal transition [45].

Cumulative social disadvantage has also been associated with an earlier age of menopause [8,34], and those who live in a negative psychosocial environment have increased reporting of symptoms [35]. Furthermore, women with a lower educational attainment have been linked with an increase in symptoms [1,28]. These factors are all relevant to Indigenous Australian women.

### Experiences in other countries and cultures

Menopause should be approached with in a biocultural paradigm because if menopausal symptoms were due solely to hormonal changes then the menopausal experience would be much more homogenous. Yet as stated previously, there are multiple factors which may influence this experience; the interaction of culture, genetics, social factors and the environment/context determines the individual woman’s experience of menopause. While country of residence does not represent universal culture, it can act as a proxy cultural measure to look at the variation in menopausal experience.

A large proportion of literature looking at cultural influences on menopausal experience has focused on Japan [18,21,29,30,38,48], often comparing it to a Western country. This interest is stimulated by the fact that Japanese women are some of the healthiest in world, outliving women in the west by five years [18]. They have a lower occurrence of osteoporosis, breast cancers, heart disease and reproductive cancers [18,39]. It is easy to assume that these differences are solely due to genetic factors, however, migration studies have shown that when Japanese women move to the United States of America their prevalence of these disorders increases to a similar rate of Caucasian American women [39]. This change in disease prevalence suggests the importance of cultural and environmental factors to the health of women [39].

In general, when compared to Western countries Asian countries have a much lower prevalence of reported vasomotor symptoms [22,26,28,44], which may be used as a surrogate measure for differences in menopausal experience. In Japan, the prevalence of hot flush reporting ranges from 37% [30] to an upper limit of 52% [48] in menopausal women. This prevalence of hot flushes is increasing; a possible reflection of the growing westernisation of Japan [30] and again highlighting the important role of culture in the health of women.

A study of Turkish women found the average age of natural menopause was 52.9 years, similar to Western women [49]. The most common symptoms were muscle, joint or bone pain (82.3%) and hot flushes (73.9%) [49]. In regards to attitudes, these women expressed a mixture of both positive and negative responses [49]. The menopausal transition was negatively viewed as “the end of youth” by 90% of women [49]. The results also showed that women perceive menopause as a heavy burden and a problem which must be finished as quickly as possible [49]. On the other hand, women viewed the actual cessation of menstruation as a positive event due to freedom from feminine hygiene products, contraception, pregnancy, and the “end of uncleanness” [49]. These findings demonstrate that there are often conflicting views regarding what menopause mean, with recognition of both positive and negative aspects to the transition [49].

Native American Indians view the menopausal transition as a neutral or positive experience and post-menopausal women are considered ‘women of wisdom’ within their communities. [20] In Canada, the average age of menopausal onset in non-Indigenous women is 51 years [23], comparable to Australia. A literature review of Canadian Aboriginal women’s experiences of menopause showed that for this ethnic group the transition was perceived as a positive experience as it had little effect on their lives except to increase social freedom [17]. While Aboriginal Canadian women had lower reporting of vasomotor symptoms, when compared to non-Aboriginal Canadian, this did not appear to be solely biological but rather an interplay of culture and tradition with biology [17]. In Australian Indigenous culture, older women are granted an increased level of respect[50], the influence of this on menopausal perception is unknown.

In a study of Mayan Indian women from Mexico, the average age of menopause was 44.3 years [39] with no participants reporting any menopausal symptoms, and the only noted event being the cessation of menses [45]. One hypothesis for this is that these women spend most of their lives pregnant or breast feeding, resulting in a constant state of low levels of circulating estrogen [36]. Thus these women may not be as greatly affected by the oestrogen withdrawal experienced at menopause [36]. Given the high parity is also very common in Mayan women loss of fertility is not a significant concern [45]. The higher parity in Indigenous women compared to non-Indigenous Australian women may indicate low levels of oestrogens and less withdrawal symptoms experienced at menopause may occur also. In addition Mayan women also face very strict restrictions whilst menstruating, with activity and food taboos. Therefore, menopause allows greater freedom and is positively anticipated by pre-menopausal women [45].

Old age is valued in contemporary Greek culture and menopause is seen as a natural transition [45]. Yet paradoxically menopause is not welcomed due to an association with growing old and being ‘out’ of mainstream of society [45]. Even though menopause is associated with the removal of a number of social taboos, for example postmenopausal women are allowed to fully participate in church activities, it is still perceived negatively [45]. Associated with these attitudes 73% [45] of Greek women reported hot flushes. Thus, while both Mayan Indian and Greek women experience the removal of menstrual taboos with menopause, their menopausal experiences are vastly different [45]. This implies that some of the freedoms granted by menopause are insufficient to explain differences in menopausal experience between cultures [45].

Maori women of New Zealand also experience similar health disparities as Australian Indigenous people when compared to the non-Indigenous women. As with Indigenous Australians, there has also been limited research on Maori women’s experiences of menopause. What is known is that the mean age of menopause is 46 years [24] for Maori women and 47 years[24] for non-Maori women. Maori women report similar rates of symptoms to non-Maori women [24].

### Indigenous Australian women

Our search strategy located only two reports on research which had explored Australian women’s experiences of menopause [36,37] (Table 3). One of these studies [36] compared Indigenous and Caucasian women in far north Queensland. In this mixed methods study, the average age of onset for menopause ranged from 45.9 years (rural areas) and 46.9 years (urban areas) for Indigenous women, compared to 48.3 years in urban Caucasian women [36]. For Indigenous women in far North Queensland, the incidence of hot flushes ranged from 36% of those living in rural areas to 71.9% of those living in urban areas [37]. Furthermore, menopause was considered an economic advantage, especially for those in rural and remote areas as they no longer need to purchase female hygiene products [36].

**Table 3:** Comparison between the two studies on menopause in Indigenous Australians

**First Author and Date**	***Davies SR et al. *Published in 2003 Research undertaken in 1999**	***McKenna, Elizabeth M *Thesis submitted in 2001 Research undertaken in 2000**
**Title**	*Climacteric symptoms among indigenous Australian women and a model for the use of culturally relevant art in health promotion *[37]	*The Experience, Knowledge and Relevance of Menopause to Indigenous and Caucasian women in Far North Queensland *[36]
**Study Design**	Cross-sectional design, using structured interviews	Face-to-Face interviewing: using both closed and open questioning technique.
		Mixed methods of analysis
**Sampling and Sample Size**	Convenience sampling in the community setting	Population based sample of women over 40 years.
	55 Participants	Snowball sampling of rural Indigenous women. Word of mouth sampling recruitment of urban Indigenous women
		Random sampling using electoral database for urban Caucasian women.
		313 Participants
		- 130 rural Indigenous women
		- 73 Indigenous women in Cairns (urban)
		- 120 Caucasian women in Cairns (urban)
**Location**	Kimberley region of Western Australia and south western Victoria - Australia	Far North Queensland - Australia
**Objective of Study**	“To evaluate climacteric symptoms among rural and remote Indigenous Australian women and to develop culturally relevant women’s health midlife educational material [37]	To investigate the knowledge and experience of menopause in Far North Queensland Indigenous women, with comparison to a Caucasian population in the same area
**Age of Menopause**	Not reported	Rural Indigenous: 45.9 years
		Urban Indigenous: 46.9 years
		Urban Caucasian: 48.3 years
**Symptoms Described**	1. Hot flushes (59%)	Lower rates of symptom reporting in the rural Indigenous women compared to the other populations in this study
	2. Urinary frequency/incontinence (53%)	
	3. Mood swings (47%)	
	4. Vaginal dryness (41%)	1. Hot flushes/night sweats
		- Rural Indigenous 36%
		- Urban Indigenous 71.9%
		- Urban Caucasian 68%
		2. Vaginal dryness
		- Rural Indigenous 29.1%
		- Urban Indigenous 56.3%
		- Urban Caucasian 46%
		3. Mood changes
		- Rural Indigenous 37.2%
		- Urban Indigenous 65.6%
		- Urban Caucasian 42%
		4. Insomnia
		- Rural Indigenous 16.3%
		- Urban Indigenous 43.8%
		- Urban Caucasian 34%
**Key Themes**	Lack of understanding about the cause of their symptoms	In rural Indigenous women, 58.9% were not aware that menses would cease.
	No traditional methods used to deal with bothersome symptoms	Celibacy at menopause was suggested by 81.5% of Indigenous women interviewed
	No use of hormone replacement therapy	Rural Indigenous women were less likely to access medical care, and to talk about menopause
		Main source of information – health professionals for Indigenous women and media for Caucasian women

In Indigenous culture, where elders are respected, the menopause transition was reported to be associated with a gain of status. [50] However, unlike menarche it does not appear to be a culturally significant event [36]. In Indigenous language groups of far north Queensland, there is no single word to correspond to ‘menopause’, rather terms such as ‘bleeding no more’ and ‘no more women’s sickness’ are used [36]. The only activity that seemed culturally dictated among rural and remote Indigenous women living in this area was the decision to become abstinent once they became postmenopausal [36]. In both of the studies identified, there seemed to be limited knowledge and understanding about menopause in general [36,37]. Indigenous women, particularly in rural or remote areas were less likely to have heard the word menopause before, and 58.9% of the Indigenous participants in the far north Queensland study were not aware their menses would cease [36]. Some of this may be attributed to the fact that ‘women’s business’ within Indigenous culture is extremely private [37]. Thus women may not recognise symptoms as being connected to the cessation of menses, thus explaining why they seek treatment for menopause at a much lower rate than Caucasian Australians [37].

## Discussion

Research so far conducted on Australian Indigenous women’s experiences of menopause has provided some insight into some of the differences between the Indigenous and non-Indigenous experience. However, the research has been reported for only two relatively specific populations. This is a significant gap in knowledge and understanding of Indigenous women and ageing considering the huge diversity between language and cultural groups across Australia. Thus, while the results provide some understanding of Australian Indigenous women’s experience of menopause, they cannot be generalised to the whole Indigenous population.

It could be hypothesised that a high average body mass index, low levels of physical activity, a higher prevalence of health risk factors such as smoking and cumulative social disadvantage in Indigenous Australians will correspond to a higher rate of menopausal symptoms, particularly vasomotor symptoms. With increasing reporting of menopausal symptoms in Japanese women with westernisation, it could also be hypothesised that a similar change to the Western lifestyle in Indigenous Australians could predispose Indigenous women to report a higher rate of symptoms. Alternatively, the higher average parity rate might help reduce symptom reporting. Further clinical and population based studies are needed to assess which of these hypotheses have validity.

While we can extrapolate the menopausal experience through various social and biological factors, there are still many questions yet to be answered. There is no consensus as to how Indigenous Australian women refer to this transition, whether there are specific words in Indigenous languages or a lack of language to describe it. There is limited understanding about Indigenous women’s traditional cultural perspectives of menopause and how culture and acculturation influences their views. Whether for Indigenous women menopause is seen as something that needs fixing or a natural transition is likely to have a major influence on their overall experience of menopause. It is also not known whether it is influenced by particular co-morbidities and the age at which it commonly occurs, or how Indigenous women cope with menopause as the limited information available suggests that they do experience menopausal symptoms. Questions remain as to what remedies are sought when there are disabling symptoms, the extent to which Indigenous culture treats this transition as a highly private experience and whether it is something that is discussed within peer groups or with health professionals.

## Conclusion

Women around the globe have diverse experiences of menopause based on various biological, psychological, social and cultural factors which shape their perception, values and attitudes to menopause. We were surprised at the dearth of studies on Indigenous Australian women and menopause, with only two studies identified. It is appropriate to ask about Indigenous women’s health seeking behaviour in relation to menopause and also to question the influence of culture, poor health including stress, and lower education literacy on their menopausal experience. This review has highlighted the lack of information on Indigenous women’s menopausal experience and explored why there is value in understanding their menopausal transition as part of providing better culturally directed health care for Indigenous Australian women. Given that modifiable lifestyle factors that contribute to poor health often are associated with increased menopausal symptoms, addressing the management of menopausal symptoms and their severity could assist Indigenous women as they age and provide another angle for health promotion and appropriate social support for this population.

## Competing interests

The authors declare that they have no competing interests.

## Authors’ contributions

EKJ and JRJ participated in the design, literature searching, and drafting of the manuscript. JMK assisted with the study design and editing of the manuscript. SCT was involved in the conception, design and editing of the manuscript. All authors read and approved the final manuscript.

## Pre-publication history

The pre-publication history for this paper can be accessed here:

http://www.biomedcentral.com/1472-6874/12/43/prepub
